# Computational Evaluation of Redox Potentials of Metal Complexes for Aqueous Flow Batteries

**DOI:** 10.1002/cphc.202500046

**Published:** 2025-04-10

**Authors:** Aliyeh Mehranfar, Jenna Hannonen, Ali Tuna, Maryam Jafarishiadeh, Anniina Kiesilä, Petri Pihko, Pekka Peljo, Kari Laasonen

**Affiliations:** ^1^ Research Group of Computational Chemistry Department of Chemistry and Materials Science Aalto University P.O. Box 16100 FI‐00076 Aalto Finland; ^2^ Research Group of Battery Materials and Technologies Department of Mechanical and Materials Engineering University of Turku FI‐20014 Turun Yliopisto Finland; ^3^ Department of Chemistry and Nanoscience Centre University of Jyvaskyla 40014 Jyväskylä Finland

**Keywords:** cyclic voltammetries, density functional theory calculations, flow batteries metal‐organic complexes, redox potentials

## Abstract

Flow batteries are a promising option for large‐scale stationary energy storage, but better redox active materials are required. Computational density functional theory (DFT) approach to materials screening can identify the most promising avenues and accelerate the development of the technology. In this work, metal complexes with functionalized organic ligands are focused on. The right redox potential, good chemical stability, and high solubility are the main characters in designing a high‐performance aqueous electrolyte. Here, Fe, Ti, Mn, and Ni are studied as central metals of the complexes with two ligand classes containing N‐ and O‐ groups. The accuracy of the DFT redox potentials is compared to experiments whenever available. In addition, some cyclic voltammetry measurements are performed for Fe‐bipyridine, phenanthroline, and terpyridine complexes. The computational redox potentials for ≈180 different metal–ligand combinations are evaluated. Overall, this work presents a new insight into the design of new electrolytes for aqueous flow batteries.

## Graphical abstract

Experimental and computational screening of redox potentials of several metal complexes is done. Computationally a large set of metal and ligand combinations has been studied. M = Fe,Ni,Ti,Mn, ligands include PIB, EDTA, cathecol, terpy, DIPSO. Most of the ligands are modified with functional groups.

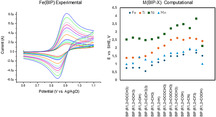

## 1 Introduction

The electrical energy produced using renewable energy sources, such as solar and wind, has increased near exponentially.^[^ ^1^ ^]^ However, many challenges remain in applying them as the main sources of energy.^[^ ^2^ ^]^ One of the core problems is the inherently large variation of the energy production, requiring integration of large‐scale energy storage to balance production and consumption of energy.

The concept of the redox flow cell as a bulk energy storage system was first proposed by Thaller in 1974.^[^ ^3^, ^4^ ^]^ The National Aeronautics and Space Administration (NASA) developed flow battery system during the energy crisis in the 1970s, and this technology has recently gained much attention as a promising candidate for stationary electrical energy storage (EES).^[^ ^5^, ^6^, ^7^ ^]^

Flow batteries have a unique configuration enabling the decoupling of energy and power. Energy storage capacity is determined by the volume of the liquid electrolyte‐containing reservoirs, while the power is determined by the area of the electrochemical cells. Electrolytes are circulated through the electrochemical cells for charging/discharging.^[^ ^8^ ^]^ The separation between power and energy components gives substantial advantages to flow batteries compared to other types of batteries and leads to great flexibility in designing a system for different applications to meet target power and energy storage capacity, especially at storage times longer than 4 h.^[^ ^9^ ^]^ Typically, water is used as an electrolyte solvent due to high ionic conductivity,^[^ ^10^, ^11^ ^]^ environmental safety, and availability. Aqueous flow batteries are inherently safe, as internal shorting will not result in uncontrollable fire like in Li‐ion batteries.^[^ ^12^, ^13^ ^]^ Vanadium‐based aqueous flow batteries are well‐established and approaching commercialization, but relatively low annual production of vanadium questions the scalability of vanadium flow batteries at the TWh scale required by the electricity systems based on renewable resources.^[^ ^14^, ^15^ ^]^ Furthermore, vanadium is listed as a critical material in Europe.^[^ ^16^, ^17^, ^18^ ^]^

The development of flow batteries based on inexpensive and sustainable redox‐active materials can overcome the drawbacks of conventional vanadium‐based flow batteries. This approach has gained increasing attention in the past few years.^[^ ^19^, ^20^, ^21^ ^]^ Many organic materials could be produced inexpensively in large quantities, and high structural tunability of the organic compounds allows tailoring the chemical and electrochemical stability, solubility, redox potential, and electrode reaction kinetics of flow batteries. Recently, some flow batteries based on organic molecules with performance comparable to vanadium‐based systems have been demonstrated, and some start‐ups are beginning to commercialize their technologies.^[^ ^22^ ^]^

Metal complex‐based batteries date back to 1979, when Adams utilized ferricyanide in the aqueous zinc–ferricyanide flow battery.^[^ ^23^ ^]^ Since then, electrochemical studies investigating transition metal complexes for example with ethylenediaminetetraacetic acid (EDTA),^[^ ^24^, ^25^ ^]^ phenanthroline,^[^ ^26^ ^]^ and diethylenetriaminepentaacetic acid (DTPA)^[^ ^27^, ^28^ ^]^ have been performed for aqueous electrolytes. A more detailed overview of different complexes is provided for example in a recent review^[^ ^29^ ^]^ by Ding et al. For example, highly soluble and stable complexes of iron with bipyridines containing carboxyl or hydroxymethyl groups^[^ ^30^ ^]^ or mixed ligands with cyanine^[^ ^31^ ^]^ for neutral pH, as well as chromium or iron complexes formed with 1,3‐propylenediaminetertaacetic acid (PDTA)^[^ ^32^ ^]^ or with 3‐[bis(2‐hydroxyethyl)amino]‐2‐hydroxypropanesulfonic acid (DIPSO),^[^ ^33^ ^]^ have been reported. For nonaqueous systems, research has focused on bipyridine, acetylacetonate, terpyridine like, and other complexes, as listed in a recent review.^[^ ^34^ ^]^

Typically, the development of novel energy storage materials relies on very time‐consuming trial and error‐based approaches. To accelerate the development, computational tools can be utilized. Computational screening approaches for flow battery chemistries have mostly focused on low‐hanging fruits such as predicting redox potentials and solvation free energies for organic molecules.^[^ ^35^, ^36^, ^37^, ^38^, ^39^, ^40^ ^]^ Redox potentials of metal complexes have also been computed by density functional theory (DFT)^[^ ^41^, ^42^, ^43^ ^]^for ≈20 complexes, but high‐throughput screening approach has not yet targeted metal complexes as active flow battery materials.

**Figure 1:**
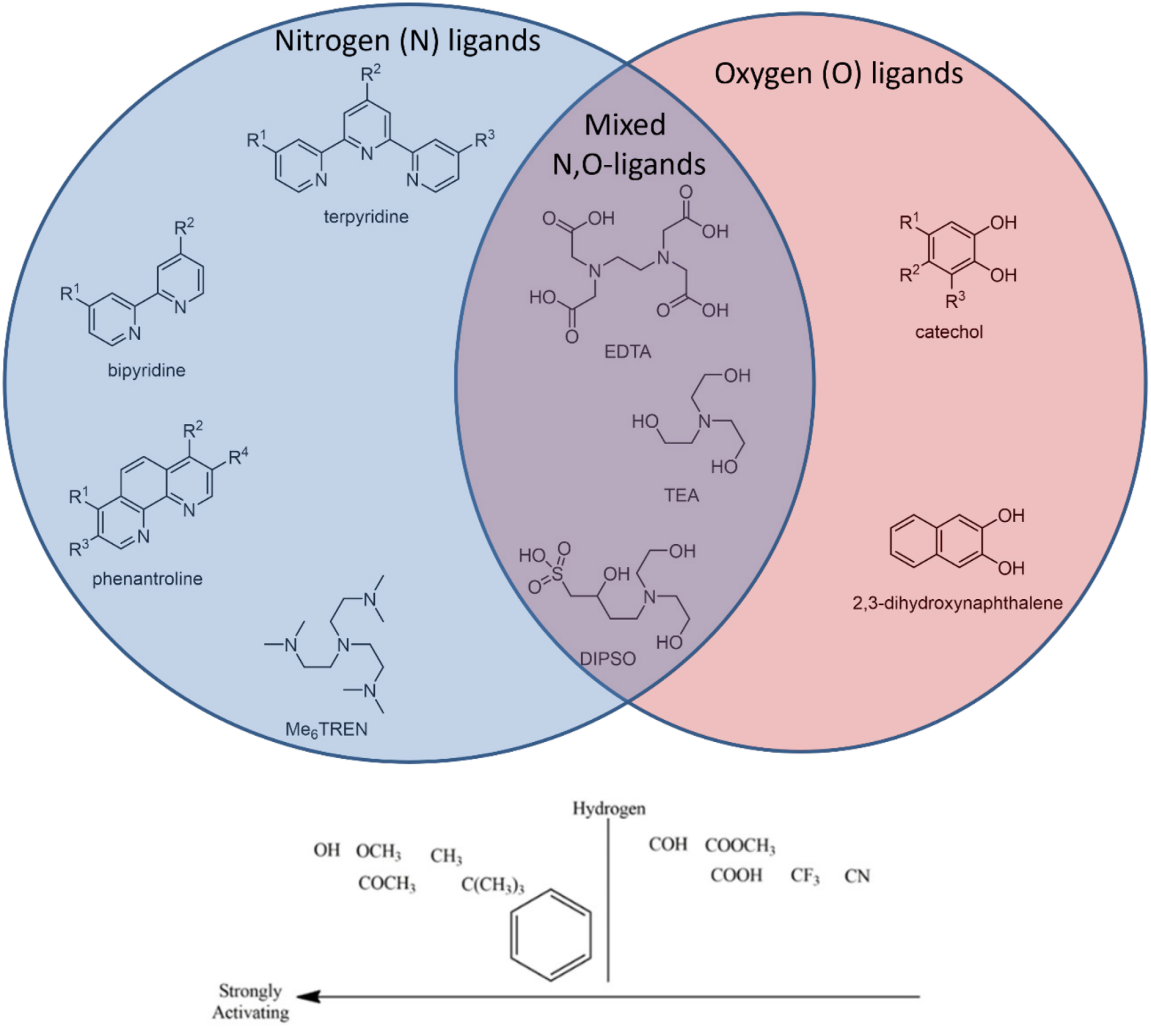
The molecular structures of the used nitrogen (N), oxygen (O), and N,O‐mixed ligands for metal complexes and functional groups used, arranged based on their activating ability.

In this article, we focus on computational modeling to screen potential metal complex candidates as redox species to realize sustainable flow batteries. We are interested in titanium (Ti), iron (Fe), manganese (Mn), nickel (Ni), and copper (Cu) as metals due to their abundance. Calculations of complexes with ligands such as EDTA, triethanolamine (TEA), tris[2‐(dimethylamino)ethyl]amine (Me6TREN), and DIPSO were compared with the available experimental data from literature or provided in this work. Calculations are focused on electrochemical properties of the proposed octahedral metal complexes. **Figure** **1** shows the selected ligand structures containing nitrogen and oxygen atoms. The parent ligands are 2,2′‐bipyridine (BIP), 2,2′:6′,2″‐terpyridine (TRP), *o*‐phenanthroline (PHEN), catechol (CAT), 2,3‐dihydroxynaphtalene (nCAT). Ligands containing aromatic structures and nitrogen were chosen as they lead to higher redox potentials.^[^ ^44^, ^45^ ^]^ The effect of different electron withdrawing and donating functional groups on computed properties was also studied. Here, the strength of electron withdrawing or donating ability could be characterized for example by effect of activation on electrophilic aromatic substitution.^[^ ^46^ ^]^ The parent ligand functionalization is performed by strong activating, strong deactivation, and moderate activating groups (Figure 1). The aim of this study is to introduce new metal‐organic redox‐active materials for designing flow batteries with appropriate redox potential, solubility. We also present some cyclic voltammetry (CV) measurements of different complexes to provide comparison for the computational values.

## 2 Experimental Section

### 2.1 Experimental Details

**Figure 2:**
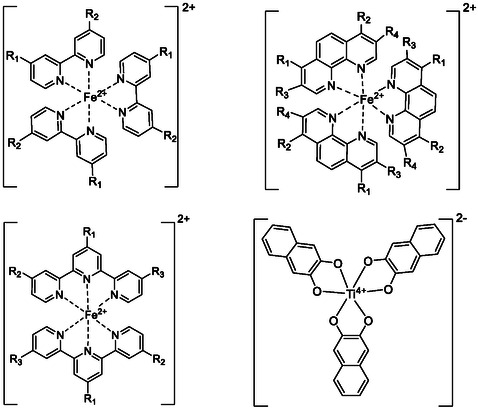
General structures of the electrochemically measured metal complexes with side chains of *R*_1_–*R*_4_.

CV measurements were performed in a three‐electrode cell: commercial Ag/AgCl with 3 m KCl as the reference electrode (BASi) and glassy carbon disk (3 mm‐diameter, BASi) as the working electrode and platinum wire as the counter electrode. The potentiostat used for measurements was Gamry Reference 600 + and aqueous supporting electrolytes are given in Supporting Information for each measurement. Metal complexes were synthesized and characterized as described in Supporting Information. The initial oxidation state of iron was +2, so most of the complexes had charge of +2, with chlorides as counter anions. The concentration of the Fe(BIP)_3_ complexes during electrochemical measurements was 0.5–0.6 mm, and for Fe(PHEN)_3_ and Fe(TRP)_3_ complexes 1 mm. Measured metal complexes included Fe(BIP)_3_ and its derivatives Fe(BIP(*R*_1,2_ = CH_3_))_3_, Fe(BIP(*R*_1,2_ = C(CH_3_)_3_))_3_, Fe(BIP(*R*_1,2_ = OCH_3_))_3_, and Fe(BIP(*R*_1,2_ = COO(CH_3_)))_3_; Fe(PHEN)_3_ and its derivatives Fe(PHEN(*R*_1,2_ = CH_3_))_3_, Fe(PHEN(*R*_1,2,3,4_ = CH_3_))_3_, and Fe(PHEN(*R*_1,2_ = Cl))_3_; Fe(TRP)_2_ and its derivatives Fe(TRP(*R*_2_ = COOH))_2_, Fe(TRP(*R*_1,2,3_ = COOH))_2_, Fe(TRP(*R*_2_ = 4py))_2_, and Fe(TRP(*R*_2_ = Cl))_2_ ; and Ti(nCAT)_3_. **Figure** **2** shows the general structures of the experimentally studied complexes with side chains of *R*_1_–*R*_4_. Experimental *E* ^0´^ values ($E_{\text{exp}}^{o}$) of these complexes were calculated as an average value of the oxidation and reduction potentials of the Fe^2+^/Fe^3+^ or Ti^3+^/Ti^4+^ redox peaks seen in the measured cyclic voltammograms (Figure S2–S6, Supporting Information).

### 2.2 Computational Details

#### 2.2.1 The Redox Potential

**Scheme 1:**
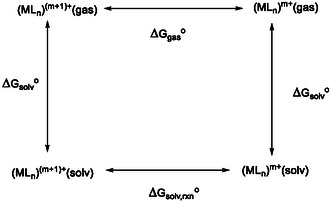
Thermodynamic cycle for the calculation of the first‐reduction potential in the gas phase and solution for the metal complexes

The free energy $\Delta G_{\text{solv,rxn}}^{o}$ and resulting electrochemical potential values of metal complexes were computed according to the thermodynamic cycle illustrated in **Scheme** **1**,^[^ ^47^ ^]^ considering the total energy of the molecule and its solvation energy $\Delta G_{\text{solv}}^{o}$ for oxidized (ML_*n*_)_gas_^(*m* + 1)+^ or reduced (ML_*n*_)_gas_^*m*+^ species. The solvation energy is computed using conductor‐like polarizable continuum model (CPCM) + SMD solvation model developed by Marenich et al.^[^ ^48^ ^]^ These two molecules also have different Gibbs free energies related to electronic total energy and the translational, rotational, and vibrational (TRV) degrees of freedom. The TRV correction to the total energy is computed in the gas phase by performing the frequency calculations in Orca software. The total free energy difference between these molecules is $\Delta G_{\text{gas}}^{o}$. With this information, we can compute the $G_{\text{solv,rxn}}^{o}$ as

(1) $$\Delta G_{\text{solv},\text{rxn }}^{o}=\;\Delta G_{\text{gas}}^{o}+\;\Delta G_{\text{solv }}^{o}{\text{(MLn)}}^{m+}-\;\Delta G_{\text{solv }}^{o}{{\text{(ML}}_{n})}^{\left(m+1\right)+}$$

Alternatively, we can compute the

(2) $$\Delta G_{\text{solv},\text{rxn }}^{o}=\Delta G_{\text{vib},\text{gas}}^{o}+\;\Delta G_{\text{tot},\text{solv }}^{o}{{\text{(ML}}_{n})}^{m+}-\Delta G_{\text{tot},\text{solv }}^{o}{\left({\text{ML}}_{n}\right)}^{{\left(m+1\right)}^{+}}$$

where the $\Delta G_{\text{tot,solv}}^{o}\left(X\right)$ is the total energy of molecule *X* using the solvation model and the $\Delta G_{\text{vib,gas}}^{o}$is the difference between the TRV corrections between the molecules (in gas phase).

From this free energy difference, the redox potential can be computed as

(3) $$E_{{\text{ML}}_{n},\text{sol}}^{o}=\frac{\Delta G_{\text{solv},\text{rxn }}^{o}}{F}-E_{\text{ref}}^{o}$$

where *F* is the Faraday constant.

The absolute reduction potential value for standard hydrogen electrode (SHE) potential reported in the literature varied from 4.05^[^ ^49^ ^]^ to 4.7 V.^[^ ^50^ ^]^ The most common reference $E_{\text{ref}}^{o}$ values were 4.28^[^ ^51^, ^52^ ^]^ and 4.44 V^[^ ^53^, ^54^ ^]^ based on omitting and including the surface potential in the solvation model, respectively. In this work we used the recommended IUPAC value for the absolute electrode potential of the hydrogen electrode 4.44 V^[^ ^55^ ^]^ at 298.15 K.

#### 2.2.2 Quantum Chemical Computations

Most of the DFT calculations were performed using the ORCA quantum chemistry program package (Version 4.0.1.)^[^ ^56^, ^57^ ^]^ The def2‐SVP adaptations of Karlsruhe basis sets were used for all the computations.^[^ ^58^ ^]^ It was reported that that def2‐SVP basis set usually was sufficient for qualitatively correct results.^[^ ^59^ ^]^ The initial octahedral complexes were constructed from crystallography data available in the literature.^[^ ^60^, ^61^, ^62^, ^63^, ^64^ ^]^ Geometry optimizations were carried out by applying the Perdew–Burke–Ernzerhof (PBE)^[^ ^64^ ^]^ functional whereas PBE0 functional was applied to assess the frequency and the single point calculations. The use of PBE0 model was not fully compatible with the structural optimization but the effect was very small. The term $\Delta G_{\text{vib,gas}}^{o}\;$ in Equation (2) was small, around 0.02–0.04 eV. So even if it were left out, our results would not change qualitatively. In some cases, PBE showed relatively large errors in calculating the total energies.^[^ ^65^ ^]^ Therefore, the hybrid form PBE0 was suggested to improve the accuracy of the calculations.^[^ ^66^ ^]^ All final structures were true energy minima since the frequency analysis showed no imaginary frequency. According to the electron configuration for octahedral *d* ^*n*^ transition‐metal complexes, Fe and Mn can have both high and low spin states. Here, the metals were generally considered in high‐spin state, due to the ligand‐field trends based on the *π*‐accepting and *π*‐donating ability of metals and ligands.^[^ ^67^ ^]^ As an example, in the case of 2,2′‐bipyridine (BIP) and 1,10‐phenanthroline (PHEN) complexes, the ligand‐field splitting in metal complexes was increased because the ligand acted as *π*‐acceptor which led to the lower energy of *t*_2g_ orbitals of metal.

The solvation effects were taken into account using the CPCM + SMD solvation model to describe the aqueous environment.^[^ ^48^ ^]^ The SMD method is more accurate compared to CPCM model due to the cavity‐dispersion contribution. (Some computations were done with Orca Version 5.0.3 and to be compatible with Version 4.0.1 ‘surfacetype gepol ses’ keyword was used.)

The solvation free energy values can be calculated using the CPCM‐SMD solvation model according to the following equation^[^ ^68^ ^]^

(4) $$\Delta G_{\text{solv}}^{o}=\Delta G_{\text{ENP}}+\Delta G_{\text{CDS}}+\Delta G_{\text{conc}}^{o}$$

where Δ*G*_ENP_ is an electrostatic term and Δ*G*_CDS_ and $\Delta G_{\text{conc}}^{o}$ are cavity‐dispersion terms and concentration change, respectively. Because $\Delta G_{\text{gas}}^{o}$ were usually calculated with the standard state of an ideal gas at 1 atm and $\Delta G_{\text{solv}}^{o}$ were calculated with the standard state of 1 m, a conversion of an ideal gas from 1 atm (24.46 mol L^−1^) to 1 m (1 mol.L^−1^) was applied with $\Delta G_{\text{conc}}^{o}=-T\Delta S^{0\to *}$ = *RT* ln (*V*_0_/V*) = *RT* ln (24.46) = 7.91 kJ mol^−1^. Therefore, the solvation energy represents the change in free energy for transfer of 1 mol of solute from the gas phase to the aqueous phase at a standard state of 1 m.^[^ ^69^ ^]^

The accuracy of DFT redox potential values obtained through this approach were confirmed recently^[^ ^70^, ^71^, ^72^, ^73^, ^74^ ^]^ and we added some additional validation computations to the Supporting Information. The redox potentials of known phenazines as well as M(BIP)_3_, (BIP = bipyridine, M = Fe,Ni,Mn) are shown in Supporting Information. Various DFT methods and def2‐TZVP basis were tested. A comparison of calculated values from the def2‐SVP basis set confirmed that the first one was sufficient to yield redox potential trends as shown in Table S3, Supporting Information. The redox potential was quite sensitive to the DFT methods used. Our choice of PBE0 methods was motivated by the good agreement with experimental Fe complexes. There are metal complexes where the hybrid functionals do not work very well^[^ ^74^ ^]^ and careful testing of the functionals is needed before doing large‐scale computations.

## 3 Results and Discussion

### 3.1 Redox Potentials of Metal Complexes

**Figure 3:**
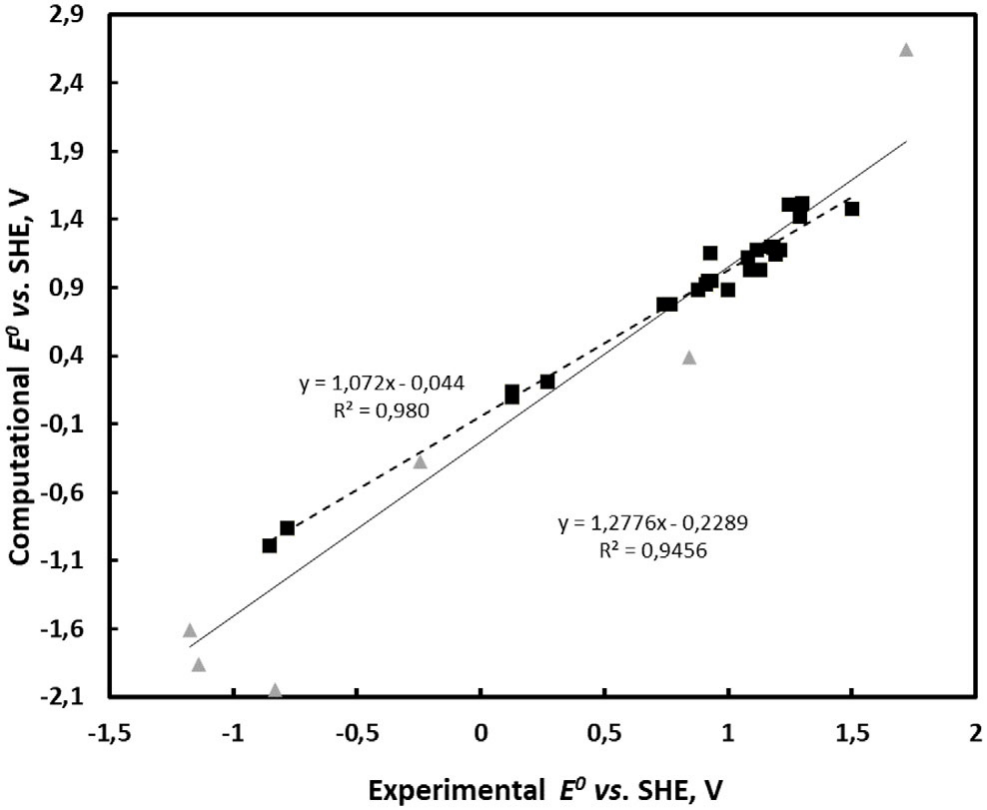
Comparison of experimental and computational redox potentials, considering only iron complexes (black squares) and all data in Table 1, including Ni, Mn, and Ti complexes (gray triangles).

Computational and experimental redox potentials of various metal complexes are presented in **Table** **1**, including some experimental values measured in this work. Most of the data is for Fe complexes. In general, the experimental and computational values listed in Table 1 align well, as shown in **Figure** **3**. The experimental CV measurements (See Supporting Information for details) for iron complexes in aqueous solutions showed expected redox pair at positive potentials, although for the Fe(BIP(*R*_1,2_ = COO(CH_3_)))_3_ only irreversible oxidation is visible. Upon initial investigation, Fe(BIP(*R*_1,2_ = COO(CH_3_)))_3_ presented two redox pairs at much lower potentials compared to iron tris‐bipyridine and no peaks at the positive potentials, shown in Figure S3, Supporting Information. However, irreversible oxidation at ≈1.5 V versus SHE was visible when scanning a wider potential range, in agreement with the computational values. The response at –0.745 V is likely iron deposition, and the remaining redox event might be due to some redox reaction in the ligand or some side product following irreversible oxidation.

**Table 1:** Experimentally determined standard potentials for the studied complexes. Please note that the oxidation state of iron, nickel and manganese is +2 to +3, and for titanium this is +3 to +4, so most of the complexes have charge of +2 and +3.

Complex		$E_{\text{DFT}}^{o}$ (V vs SHE)	$E_{\text{exp}}^{o}$ (V vs SHE)	References
Fe(TRP)_2_	6N	1.027	1.130 1.089	This work [79]
Fe(TRP(*R*_2_ = COOH)_2_	6N	1.20, protonated	1.173	This work
Fe(TRP(*R*_1,2,3_ = COOH)_2_	6N	1.51, protonated	1.245	This work
Fe(TRP(*R*_2_ = 4py)_2_	6N	1.12	1.185	This work
Fe(TRP(*R*_2_ = Cl)_2_	6N	1.37	1.191	This work
Fe(PHEN(*R*_1,2,3,4_ = CH_3_))_3_	6N	0.884	0.879 1.00	This work [80]
Fe(PHEN(*R*_1,2_ = CH_3_))_3_	6N	0.944	0.920 0.934	This work [80]
Fe(PHEN)_3_	6N	1.178	1.115 1.209	This work [81]
Fe(PHEN(*R*_1,2_ = Cl))_3_	6N	1.37	1.290	This work
Fe(EDTA)	3O 2N	0.136	0.125	[75]
Fe(EDTA).H_2_O	3O 2N	0.097	–	–
Fe(EDTA)	4O 2N	−0.718	–	–
Fe(EDTA).H_2_O	4O 2N	−0.716	–	–
Fe(TEA)	3O 1N	−0.858	−0.780	[82]
Fe(DIPSO)	3O 1N	−0.990	−0.851	[33]
Fe(DIPSO). H_2_O	3O 1N	−0.911	–	–
Fe(PDTA)	3O 2N	0.212	0.269	[75]
Fe(BIP)_3_	6N	1.122	1.080 1.030	This work [30]
Fe(BIP(*R*_1,2_ = CH_3_))_3_	6N	0.923	0.910	This work
Fe(BIP(*R*_1,2_ = C(CH_3_)_3_))_3_	6N	1.15	0.930	This work
Fe(BIP(*R*_1,2_ = OCH_3_))_3_	6N	0.779	0.765	This work
Fe(BIP(*R*_1,2_ = COO(CH_3_)))_3_	6N	1.475	≈1.5 V; +0.016 V; −0.745 V	This work
Fe(BIP(*R*_1,2_ = COO(CH_3_)))_3_	4O	−3.138	–	–
Fe(BIP(*R*_1,2_ = COO(CH_3_)))_3_	2O 2N	−1.516	–	–
Fe(BIP(*R*_1,2_ = O^−^)_3_	6N	0.779 (protonated)	−0.053 V (pH 12, deprotonated)	[30]
Fe(BIP(*R*_1,2_ = OCH_3_))_3_	6N	0.779	0.74	–
Fe(BIP(*R*_1,2_ = COOH))_3_	6N	1.518 (protonated)	1.30 1.175	[30] This work
Fe(CAT)_3_	6O	−2.049	−0.83	[43]
Ni(BIP)_3_	6N	2.647	1.72	[76]
Mn(EDTA)	4O 2N	0.391	0.841	[83]
Mn(TEA)	3O 1N	−0.374	−0.245	[84]
Ti(CAT)_3_	6O	−1.86	−1.175	This work
			−1.139	[85]

Note that we have four theoretical values for Fe(EDTA) in Table 1. The EDTA could coordinate to Fe with five dentate (2N, 3O), redox potential 0.136 V, or six‐dentate coordination (2N, 4O), potential −0.718 V. The experimental value is 0.125 V^[^ ^75^ ^]^ which agrees very well with the five coordinated structures. These results show that the redox potential of the complex is very sensitive to the structure of the metal complex. Good agreement with the DFT results and experiments is very likely indicating that the structure used in computations is the same than in the actual experiments. Therefore, the comparison of the computational and experimental redox potentials can be used to evaluate the structure of the metal complex in solution. We also added explicit water molecule to some of the calculations, but this did not change the redox potentials.

**Figure 4:**
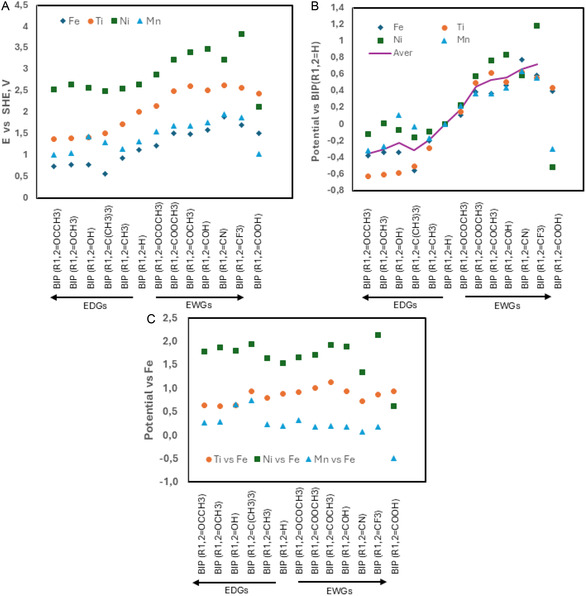
A) The redox potential versus SHE for complexes of tris‐bipyridine and its functionalized derivates for proposed metals Ni, Ti, Mn, and Fe. B) The potential difference versus parent bipyridine (BIP (*R*_1,2_ = H)) for different metals. C) The effect of metal on the redox potential presented by the potential difference versus iron complex.

Figure 3 shows that experimental and computational values agree very well for iron complexes, but unfortunately, there is not much data available for other metals in the aqueous environment. Computational value for titanium catechol (CAT) complex is 700 mV lower than experimental values, and the difference with iron catechol is 1200 mV. The computational difficulty of this complex was also noted earlier.^[^ ^43^ ^]^ Mn(EDTA) shows too low computational value can be due to wrong coordination, while Mn(TEA) is in good agreement. Ni(BIP)_3_ has experimentally reported potential of 1.72 V in a review,^[^ ^76^ ^]^ but the source of this value is not clear. We did a detailed comparison of the non‐Fe results, and they are in the Supplementary Information. The verifications of the accuracy of the computed results of Ti, Cu, Ni, and Mn would greatly benefit of additional experimental values. We do not have experimental values for Cu complexes and the Cu complex values were left out from this article. Our predictions of the absolute redox potentials of non‐Fe systems (**Figure** **4**a,c) are far from certain. The Mn values are the most reliable, but Ni values can be even 1 V too high. The relative values in Figure 4b should more reliable.

In order to identify the influence of the functionalization of parent ligands, the redox potential values for proposed complexes with functionalized ligands (Figure 1) are calculated and summarized in a Supplementary Excel file. Computationally expensive frequency calculations were not performed for all the functionalized complexes as the difference of free energy correction for the functionalized and parent complexes was negligible (less than 0.1 eV). Therefore, $\Delta G_{\text{vib},\text{gas}}^{o}$ of the parent complexes are used for the whole functionalized family instead of individual values of the functionalized complexes.

Figure 4 summarizes the redox potentials for different metals as well as effects of different functional groups for different bipyridine complexes of Ti, Mn, Fe, and Ni. Only tris‐complexes were considered, as they are known for Mn, Fe, and Ni. For example copper is known to make mono, bis, and tris complexes.^[^ ^77^ ^]^ The redox potentials of complexes decrease from Ni, Ti, Mn to Fe with Ni complex at *≈*1.68 V higher potential than Fe complex. The differences for Ti, and Mn are 0.84, and 0.23 V, respectively. In general, electron withdrawing groups (EWGs) increase and electron donating groups (EDGs) decrease the computational redox potentials, although some outliers exist. Interestingly, EDGs decrease potential on average by up to 350 mV, while EWGs can increase the potential by up to 700 mV. The order of the functional groups listed by the average change on the redox potential is slightly different than if ordered by the effect on activation of electrophilic aromatic substitution. Large variations are observed with –COOH group also for other types of ligands (Figure S7, Supporting Information). EDGs have the most effect on Ti complexes and the least effect on Ni complexes.

The values of theoretical solvation free energies and some discussion of them are in the Supporting Information. The results are quite obvious, the high charge complexes have higher solvation energy. A more detailed discussion is in the Supporting Information.

The redox potentials of all the calculated complexes are shown in Figure S7, Supporting Information.

Generally, for N‐ligands, the orders of metals based on their redox potentials are Ni (2–3.5 V) > Ti (1–3 V) > Mn (1–2 V) > Fe (0.5–2 V). For O‐ligands, the order changes to Ni, Mn, Fe, and Ti. Based on the redox potential range, some Ti, Fe, and Mn complexes with N‐ligands could be interesting as positive materials for aqueous flow batteries. Ni complexes show too high potentials for aqueous systems, but they could be suitable for nonaqueous systems that are not limited by the stability window of water. Using nonaqueous solvents with a wide electrochemical window allows a much wider selection of the redox couples, as for example, acetonitrile is stable over 5 V and widely used as a nonaqueous solvent.^[^ ^78^ ^]^ Ni, Mn, Ti, and Fe complexes with *O*‐ligands reside at the negative potential range (–0.26 to *≈*–2 V), apart from EDTA (1–0 V) and some outliers.

There are several suitable Ti complexes in aqueous media with BIP, TRP, and PHEN ligands. (Figure 4 and S7, Supporting Information). The redox potential value of Ti‐TEA is −0.932 V which makes it convenient as negative material. Ti‐EDTA complex has redox potential value of 0.362 V and Ti‐TRP complexes are 1.21–1.72 V and several Ti‐BIP complexes are below 1.8 V. In the case of Mn complexes, several BIP, TRP, PHEN, and EDTA ligands are potential electrolytes for the positive side (Figure 4 and S7, Supporting Information). The number of suitable Fe complexes as catholyte is also high. Iron complexes including BIP, TRP, PHEN, EDTA and their functionalized structures by EDGs can be considered as positive electrolytes (Figure 4 and S7, Supporting Information). The obtained results confirmed the role of EWGs to increase the redox potential values and EDGs in reducing it. Therefore, applying EWGs to parent ligands is helpful in designing high‐potential catholytes, and ligand functionalization by strong EDGs leads to low potential anolytes.

## 4 Conclusion

In this study, DFT calculations are applied to determine the main factors for designing organometallic redox flow battery chemicals. Several metals and ligands were investigated, and the ligands were modified with electron‐withdrawing or donating groups. The DFT calculations can reliably predict the redox potentials of the complexes. In addition to literature data, we performed some CV measurements to confirm the DFT predictions. We notice that the computed redox potential is very sensitive to the structure of the molecule, and we propose that this can be used to determine the structure of the metal complexes in solution environment.

By plotting the redox potentials and solvation energies of the different candidates (Figure S9, Supporting Information), we can see that Fe and Mn, as well as some Ti N‐complexes show promising properties as positive electrolytes and some O‐ligand complexes with Ni, Mn, and Fe as central metals seem to show also adequate properties as negative electrolytes, while in many cases the redox potentials are too negative. The best positive electrolyte candidates to be used in an aqueous environment can be designed based on the Fe and Mn N‐ligands complexes including weak electron withdraw groups.

Overall, the DFT computations serve as a tool for searching promising molecules for flow batteries. We can by calculations test hundreds of metal–ligand combinations and find the candidates with appropriate redox potential with maximal solvation free energy. Naturally, the DFT predictions must be tested also experimentally, but it is not realistic to perform hundreds of such experiments in practice. Thus, the approach presented in this work will clearly speed up the search for new materials for energy storage solutions.

## Conflict of Interest

The authors declare no conflict of interest.

## Supporting information

Supplementary Material
