# Supplementary material for: Computational Evaluation of Redox Potentials of Metal Complexes for Aqueous Flow Batteries
Source: Chemphyschem. 2025 Apr 10;26(12):e202500046. doi: 10.1002/cphc.202500046 (PMC12188176; doi:10.1002/cphc.202500046)
Supplement: Supplementary file 1 — Supplementary Material [file CPHC-26-e202500046-s001.zip › cphc.202500046-sup-0001-suppdata-S1.pdf]

Supplementary data

Computational Evaluation of Redox Potentials of Metal Complexes for Aqueous Flow Batteries

Aliyeh Mehranfar[a], Jenna Hannonen[b], Ali Tuna[b], Maryam Jafarishiadeh[a], Anniina Kiesilä[c], Petri Pihko[c], Pekka Peljo[b,*], Kari Laasonen[a,*]

[a]*Research Group of Computational Chemistry, Department of Chemistry and Materials Science, Aalto University, P.O. Box 16100, FI-00076 Aalto, Finland*

[b]*Research Group of Battery Materials and Technologies, Department of Mechanical and Materials Engineering, University of Turku, FI-20014 Turun Yliopisto, Finland*

[c]*Department of Chemistry and Nanoscience Centre, University of Jyvaskyla, 40014 Jyväskylä, Finland*

Chemicals and materials

The mentioned phenanthroline and terpyridine ligands (purity >97-98%), and anhydrous $FeCl_2$ (97%) in Table S1, were purchased from Fluorochem, UK. Titanium isopropoxide (97%), 25% sodium methoxide solution in methanol (98%), and 2,3-dihydroxynapthalene (98%) were purchased from TCI Europe, Netherlands. Anhydrous methanol was purchased from Acros Organic, Spain and deuterated NMR solvents (d_6-DMSO and D_2O, min 99.9 D%) were obtained from ARMAR Isotopes, Germany. The glassware and other things were obtained from VWR, Finland.

Synthesis of the metal complexes

Iron 2,2´-bipyridines were synthesized and characterized as in paper[1]. The iron complexes of phenanthroline and terpyridine derivatives were synthesized according the procedure below.

Preparation of iron complexes:

To a 100 mL beaker with a small stirring bar, ligand (3 eq for phenanthroline or 2 eq for terpyridine) was dissolved in 40-45 mL of anhydrous methanol. The solution was heated up to 60-65˚C and anhydrous ferrous chloride ($FeCl_2$, 1 eq) was added (please see the Table S0 below). The reaction mixture´s color changed immediately upon forming Fe-complexes and the mixture was heated up to 60-65˚C for additional 10 minutes. Afterwards, the reaction mixture was transferred to 100 mL of one-necked round bottom flask using funnel and the residue was washed with minimum amount of anhydrous methanol. The solution was slowly evaporated under reduced pressure to give desired Fe-complexes. The yields were either calculated before or after further drying the iron complexes in reduced pressure at room temperature to be found more than 99% complex formation. The

complexes were analyzed by ^{1}H-NMR and ^{13}C-NMR spectroscopy together with the comparison of free-base ligands in d_6-DMSO.

Table S1. Amounts of used ligands and $FeCl_2$ in synthesis.

Ligands	Ligand amount (mg)	$FeCl_2$ amount (mg)
1,10-phenanthroline	4263	1000
4,7-dimethyl-1,10-phenanthroline	912	185
3,4,7,8-tetramethyl-1,10-phenanthroline	5000	894
4,7-dichloro-1,10-phenanthroline	849	144
2,2´:6´,2´´-terpyridine	1839	500
2,2´:6´,2´´-terpyridine-4’-carboxylic acid	897	205
2,2´:6´,2´´-terpyridine-4,4’,4’’-tricarboxylic acid	1000	176
4’-chloro-2,2´:6´,2´´-terpyridine	866	205
4’-(4’’’-pyridyl)-2,2´:6´,2´´-terpyridine	999	205

$[Fe^{II}(phen)_3]^{2+}$: ^{1}H-NMR (600 MHz, d_6-DMSO, 298 K, δ ppm): 8.86 (s, 2H), 8.44 (s, 2H), 7.78 (s, 2H), 7.74 (s, 2H); ^{13}C-NMR (150 MHz, d_6-DMSO, 298 K, δ ppm): 156.32 (2C), 149.49 (2C), 137.91 (2C), 130.91 (2C), 128.54 (2C), 126.78 (2C).

$[Fe^{II}(DMe\text{-}phen)_3]^{2+}$: ^{1}H-NMR (600 MHz, d_6-DMSO, 298 K, δ ppm): 8.47 (s, 2H), 7.58 (s, 2H), 7.54 (d, 2H, J = 4.2 Hz), 2.89 (s, 6H); ^{13}C-NMR (150 MHz, d_6-DMSO, 298 K, δ ppm): 154.75 (2C), 148.65 (2C), 147.04 (2C), 129.42 (2C), 126.82 (2C), 124.57 (2C), 18.14 (2C).

$[Fe^{II}(TMe\text{-}phen)_3]^{2+}$: ^{1}H-NMR (600 MHz, d_6-DMSO, 298 K, δ ppm): 8.48 (s, 2H), 7.29 (s, 2H), 2.78 (s, 6H), 2.19 (s, 6H); ^{13}C-NMR (150 MHz, d_6-DMSO, 298 K, δ ppm): 155.46 (2C), 148.27 (2C), 145.36 (2C), 134.98 (2C), 128.81 (2C), 124.82 (2C), 18.24 (2C), 14.93 (2C).

$[Fe^{II}(DCl\text{-}phen)_3]^{2+}$: ^{1}H-NMR (600 MHz, d_6-DMSO, 298 K, δ ppm): 8.58 (s, 2H), 7.93 (s, 2H), 7.80 (d, 2H, J = 4.2 Hz); ^{13}C-NMR (150 MHz, d_6-DMSO, 298 K, δ ppm): 157.22 (2C), 149.45 (2C), 143.50 (2C), 128.41 (2C), 126.76 (2C), 125.13 (2C).

$[Fe^{II}(terpy)_2]^{2+}$: ^{1}H-NMR (600 MHz, d_6-DMSO, 298 K, δ ppm): 9.42 (s, 2H), 8.97 (s, 2H), 8.84 (s, 1H), 8.00 (s, 2H), 7.20 (s, 2H), 7.14 (s, 2H); ^{13}C-NMR (150 MHz, d_6-DMSO, 298 K, δ ppm): 160.10 (2C), 158.09 (2C), 153.06 (2C), 139.30 (1C), 138.66 (2C), 128.15 (2C), 124.74 (2C), 124.62 (2C).

$[Fe^{II}(1COOH\text{-}terpy)_2]^{2+}$: ^{1}H-NMR (600 MHz, d_6-DMSO, 298 K, δ ppm): 9.61 (s, 2H), 9.07 (d, 2H, J = 6.6 Hz), 7.99 (s, 2H), 7.23 (s, 2H), 7.16 (s, 2H); ^{13}C-NMR (150 MHz, d_6-DMSO, 298 K, δ ppm): 165.55 (1C), 160.18 (2C), 157.10 (2C), 152.75 (2C), 152.73 (1C), 139.07 (2C), 127.86 (2C), 124.60 (2C), 123.05 (2C).

[Fe^{II}(3COOH-terpy)$_2$]$^{2+}$: ^{1}H-NMR (600 MHz, d_6-DMSO, 298 K, δ ppm): 9.86 (s, 2H), 9.35 (d, 2H), 7.47 (s, 2H), 7.37 (s, 2H); ^{13}C-NMR (150 MHz, d_6-DMSO, 298 K, δ ppm): 165.98 (1C), 165.01 (2C), 160.28 (2C), 158.60 (2C), 154.59 (1C), 154.47 (2C), 141.46 (2C), 126.96 (2C), 124.82 (2C), 124.04 (2C).

[Fe^{II}(Cl-terpy)$_2$]$^{2+}$: ^{1}H-NMR (600 MHz, d_6-DMSO, 298 K, δ ppm): 9.56 (s, 2H), 8.91 (d, 2H, J = 7.7 Hz), 8.01 (t, 2H, J = 7.7 Hz), 7.31 (d, 2H, J = 5.5 Hz), 7.19 (t, 2H, J = 6.6 Hz); ^{13}C-NMR (150 MHz, d_6-DMSO, 298 K, δ ppm): 160.43 (2C), 156.78 (2C), 153.21 (2C), 144.86 (1C), 138.92 (2C), 127.91 (2C), 124.51 (2C), 124.46 (2C).

[Fe^{II}(4py-terpy)$_2$]$^{2+}$: ^{1}H-NMR (600 MHz, d_6-DMSO, 298 K, δ ppm): 9.99 (s, 2H), 9.31 (d, 2H, J = 5.8 Hz), 9.02 (s, 2H), 8.72 (s, 2H), 8.07 (s, 2H), 7.32 (s, 2H), 7.22 (s, 2H); ^{13}C-NMR (150 MHz, d_6-DMSO, 298 K, δ ppm): 160.76 (2C),158.20 (2C), 153.29 (2C), 151.41 (2C), 146.67 (1C), 143.52 (1C), 139.38 (2C), 128.24 (2C), 125.10 (2C), 122.39 (2C), 121.93 (2C).

Preparation of titanium complex:

To a 100 mL beaker with a small stirring bar, 2,3-dihydroxynapthalene (7.2077 g, 45 mmol, 3 eq) was dissolved in 50-55 mL of isopropanol. Afterwards, 25 % $NaOCH_3$ solution in methanol (18-18.1 mL, 90-91 mmol, ~6 eq) was added dropwise. The solution was heated up to 40-45˚C and titanium isopropoxide (4.44 mL, 15 mmol, 1 eq) was added dropwise. The reaction mixture´s color changed to red immediately upon forming titanium catechol complex and the mixture was stirred at 45˚C for additional 30 minutes. Afterwards, the reaction mixture was transferred to 100 mL of one-necked round bottom flask using funnel and the residue was washed with minimum amount of anhydrous isopropanol. The solution was slowly evaporated under reduced pressure to give desired sodium salt of titanium dihydroxynaphthalene complex. The complex was analyzed by ^{1}H-NMR in d_6-DMSO.

Na_2[Ti^{IV}(dihydroxynaphthalene)$_3$]] ^{1}H-NMR (600 MHz, d_6-DMSO, 298 K, δ ppm): 8.15 (dd, 2H, J^1 = 9.0 Hz, J^2 = 14.7 Hz), 8.97 (s, 2H), 8.84 (dt, 2H, J^1 = 9.0 Hz, J^2 = 14.7 Hz).

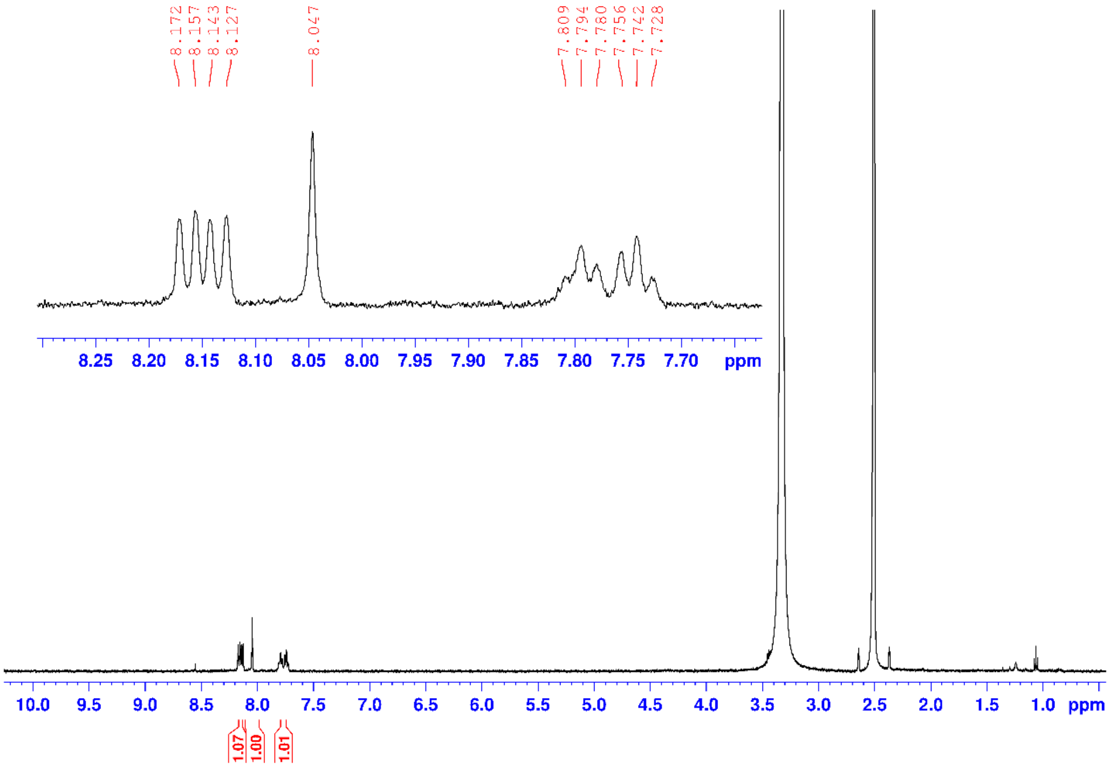

Figure S1. ^{1}H-NMR of sodium salt of titanium dihydroxynapthalene complex (Ti-nCAT) in d_6-DMSO.

Cyclic voltammograms

Cyclic voltammetry (CV) measurements were performed in a three-electrode cell: commercial Ag/AgCl with 3 M KCl as the reference electrode (BASi), glassy carbon disk (3 mm diameter, BASi) as the working electrode and platinum wire as the counter electrode. Redox potentials were converted by checking the potential of the used Ag/AgCl electrode versus a known master reference electrode, and then using the known potential difference between SHE and Ag/AgCl electrodes to convert the potential. The saturation and species of the inside solution in the Ag/AgCl electrode were considered in the conversion.

Cyclic voltammograms of the metal complexes are presented in Figures S1-S5.

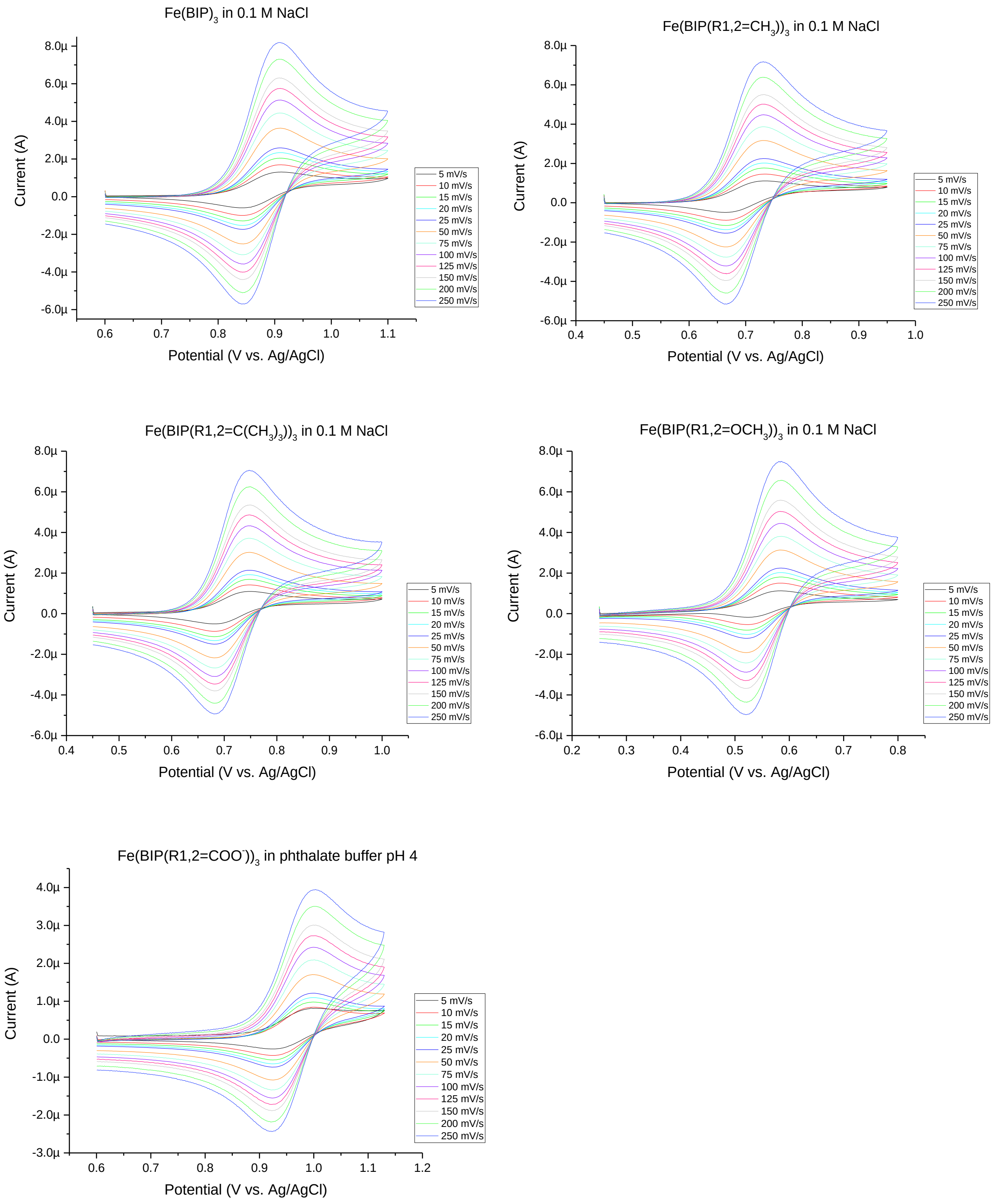

Figure S2. Cyclic voltammograms recorded for $Fe(BIP)_3$ and its derivatives $Fe(BIP(R_{1,2}{=}CH_3))_3$, $Fe(BIP(R_{1,2}{=}C(CH_3)_3))_3$, $Fe(BIP(R_{1,2}{=}OCH_3))_3$ and $Fe(BIP(R_{1,2}{=}COO^-))_3$ with different scan rates. Electrolytes given in the graphs.

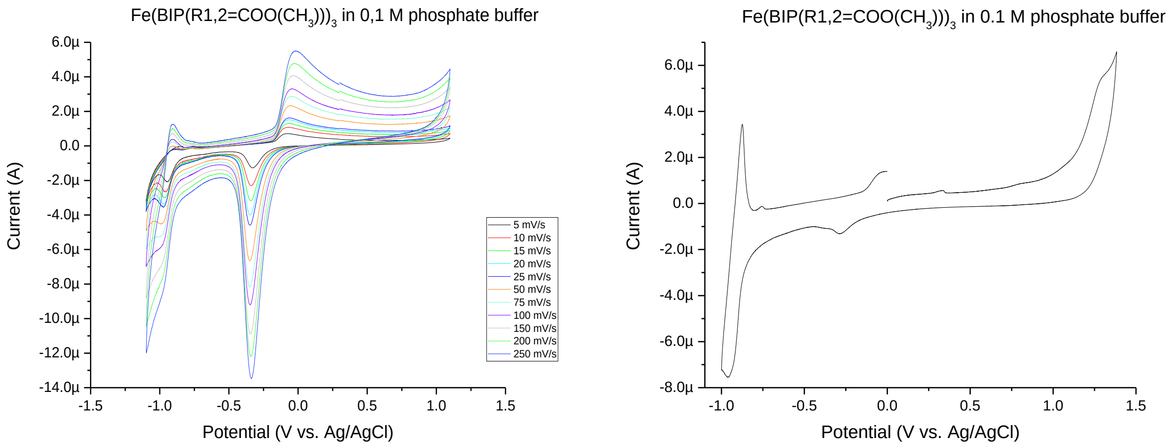

Figure S3. Cyclic voltammograms recorded for Fe(BIP($R_{1,2}$=COO(CH_3)))$_3$, up to 1.2 V (left) and up to 1.4 V (right) The Fe redox pair might originate from positive potentials with an irreversible response, since an oxidation peak is detected at 1.3 V vs. Ag/AgCl (1.5 V vs. SHE), which would correspond to the computational value (1.475 V vs. SHE).

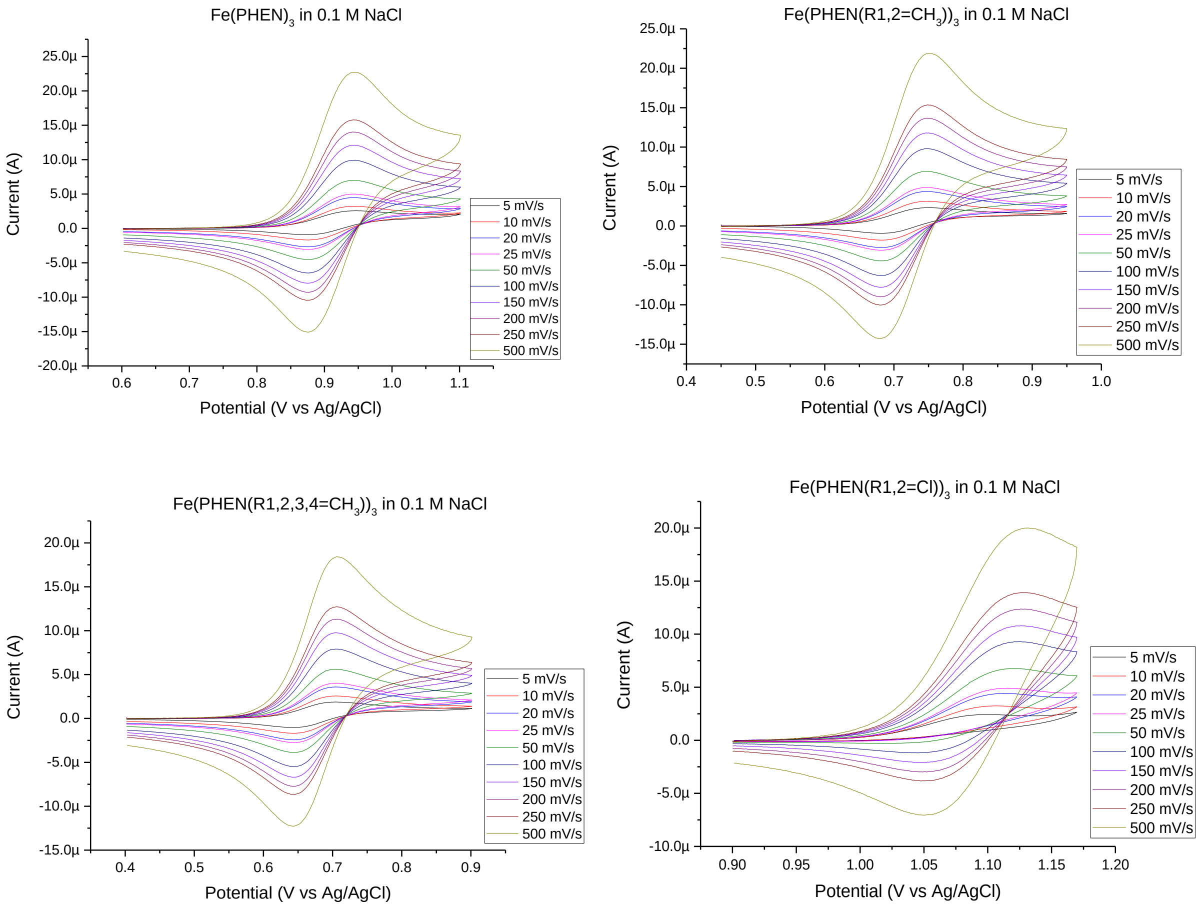

Figure S4. Cyclic voltammograms recorded for $Fe(PHEN)_3$ and its derivatives $Fe(PHEN(R_{1,2}=CH_3))_3$, $Fe(PHEN(R_{1,2,3,4}=CH_3))_3$, and $Fe(PHEN(R_{1,2}=Cl))_3$ with different scan rates in 0.1 M NaCl.

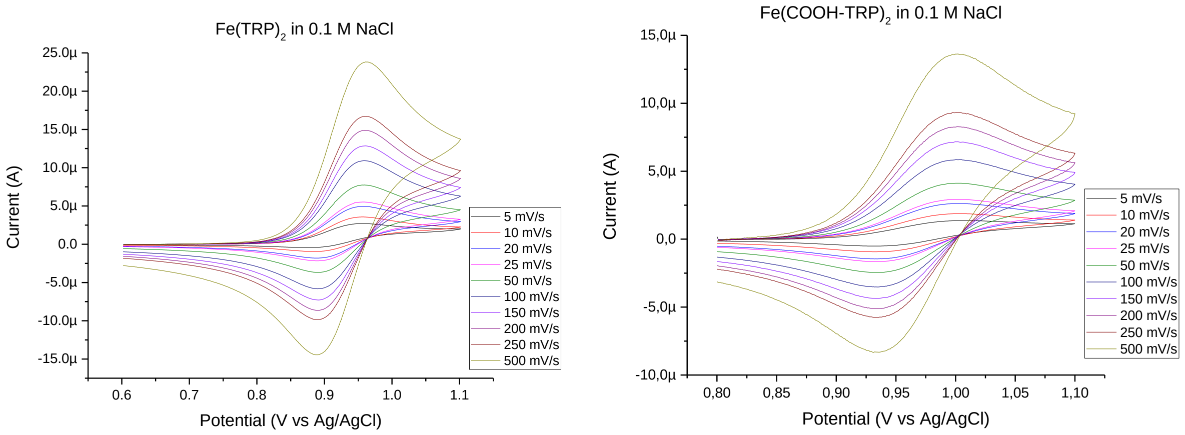

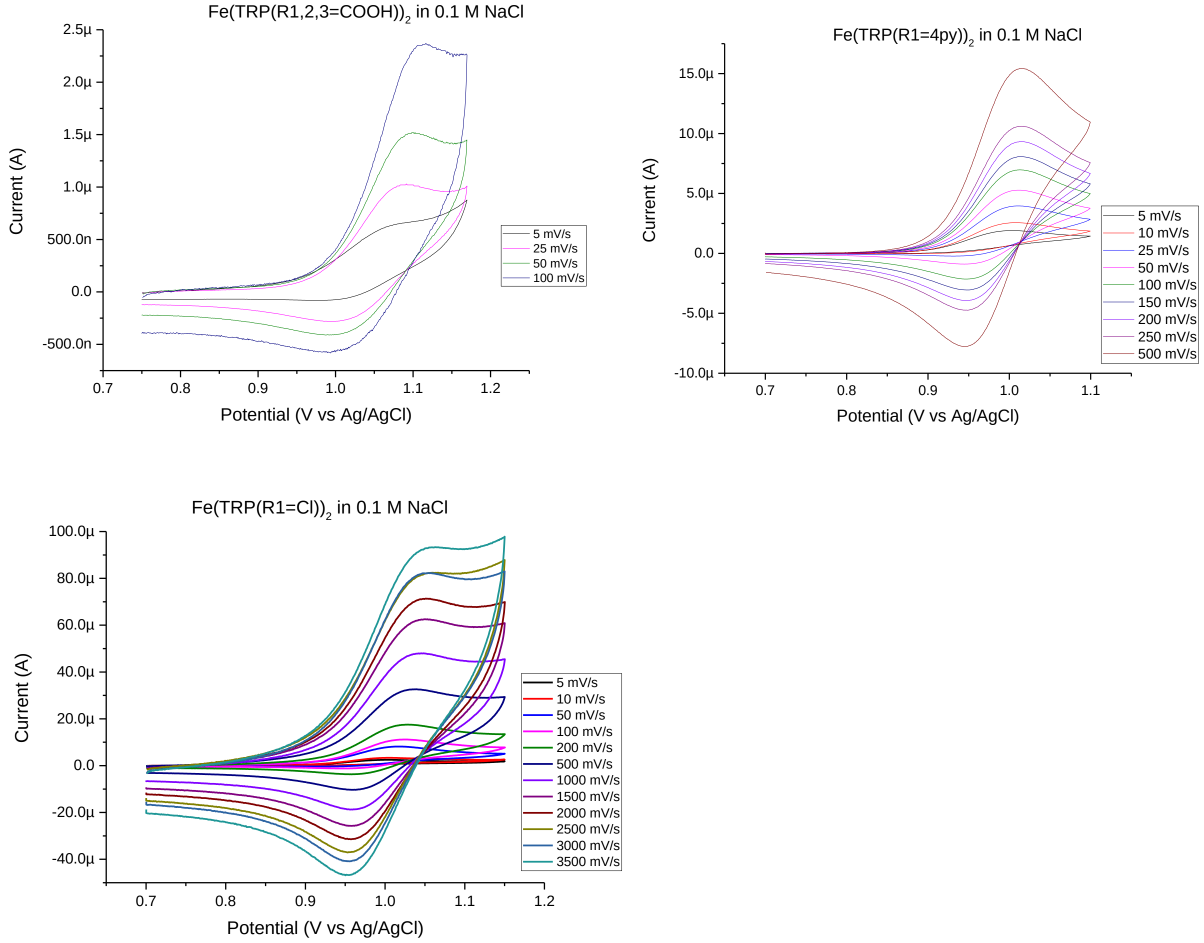

Figure S5. Cyclic voltammograms recorded for $Fe(TRP)_2$ and its derivatives $Fe(TRP(R_2{=}COOH))_2$, $Fe(TRP(R_{1,2,3}{=}COOH))_2$, $Fe(TRP(R_2{=}4py))_2$ and $Fe(TRP(R_2{=}Cl))_2$ with different scan rates in 0.1 M NaCl.

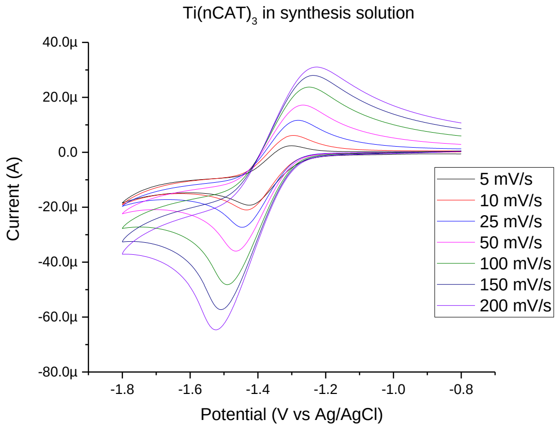

Figure S6. Cyclic voltammogram recorded for Ti(nCAT)$_3$ in the synthesis solution.

The computational approach accuracy, redox potential

First the accuracy of the computational approach is validated through the prediction of redox potential values of some phenazine-based structures and compared with earlier theoretical and experimental results from cyclic voltammetry (CV).[2,3] CV measurements were performed by using ferricenium/ferrocene (Fc^+/Fc) couple as an internal standard in dimethoxyethane (DME) solvent. The redox potential values, in Ref 2 (Computed $E_1^{\circ,a}$ (V)) were calculated by performing single-point M06-2X[4] energy calculations. The results are summarized in Table S2 and it reveals a good agreement between our and previous theoretical and experimental redox potential values, so the choice of the density-functional method and basis set is adequate.

Table S2. Comparison between the experimental values of the first-electron reduction potential for the phenazine-based compounds vs. Fc/Fc^+ in the DME solvent and the calculated values with the employed methodology in this work

Compound	Computed $\mathbf{E_1^{\circ}}$ (V) (This work)	Computed $\mathbf{E_1^{\circ,a}}$ (V)	Experimental $\mathbf{E_1^{\circ,b}}$ (V)
1,3,9-Phenazine–$(CF_3)_3$	-1.28	-1.14	-1.07
1,4,6-Phenazine–$(CF_3)_3$	-1.71	-1.13	-1.12
1,2,4,6,9-Phenazine–$(CF_3)_5$	-0.90	-0.92	-0.97
1,2,4,6,7,9-Phenazine–$(CF_3)_6$	-0.69	-0.42	-0.46
1,3,4,6,7,9-Phenazine–$(CF_3)_6$	-0.39	-0.44	-0.46

[a] Obtained from ref. 2

[b] Obtained from ref. 3

As another test, the redox potential value for Ferrocene couple (Fc/Fc^+) in an aqueous solvent was calculated. The calculated absolute reduction potential for this couple is 5.123 V, which is close to the experimental value (5.08 V)[5], the corresponding aqueous SHE potential (4.44 V) is included in absolute reduction potential values. Lastly some additional tests of basis sets, DFT methods and solvation models were done. We focused on Phenazine and $M(BIP)_3$, bipyridine=BIP molecules. The results are in Table S3.

Table S3. Comparison between the redox potential values of the first-electron reduction potential for the phenazine-based compounds vs. Fc/Fc^+ in the aqueous solvent at 6-31+G(d,p) and def2-SVP basis sets. In the Me-BIP series def2-TZVP basis was used. In all cases, the geometry was optimized with PBE model and def2-SVP basis. For Me-BIP we did not include the vibrational correction ($\Delta G^{o}_{vib,gas}$). In the BIP, PBE0 case it is 0.035 eV. For dispersion we used Grimme's D3 correction[6] with BJ-damping[7].

**Redox potentials (in eV)**	**def2-SVP**	**6-31+G(d,p)**
**Phenazine**	-1.66	-1.74
**1,3,9-Phenazine–(CF_3)$_3$**	-1.29	-1.14
**1,3,4,6,7,9-Phenazine–(CF_3)$_6$**	-0.41	-0.47
**Fe(BIP)$_3$**	**def2-SVP**	**def2-TZVP**
redox (PBE0, SMD solvation model)	1.08	1.10
redox (PBE0, SMD solvation, dispersion D3BJ)	1.07	
redox (PBE0, SMD solvation, PBE0 opt)	1.01	
redox (PBE0, CPCM solvation model)	1.19	1.10
redox(PBE, SMD solvation)	0.80	0.805
redox(M06, SMD solvation)	0.93	
redox(B3LYP, SMD solvation)	0.96	
**Ni(BIP)$_3$**	**def2-SVP**	**def2-TZVP**
redox(PBE0, SMD solvation)	2.67	2.63
redox(PBE, SMD solvation)	2.10	
**Mn(BIP)$_3$**	**def2-SVP**	**def2-TZVP**
redox(PBE0, SMD solvation)	-0.09	
redox(PBE, SMD solvation)	-0.51	
**Fe(BIP($R_{1,2}$=CH_3))$_3$** (PBE0, SMD)	0.89	0.88
**Fe(BIP($R_{1,2}$=CH_3))$_3$** (PBE0, SMD, disp D3BJ)	0.86	
**Fe(BIP($R_{1,2}$=$C(CH_3)_3$)$_3$** (PBE0, SMD)	0.60	
**Fe(BIP($R_{1,2}$=$C(CH_3)_3$)$_3$** (PBE0, SMD,D3BJ)	0.63	

The study of Toma et al. [8] is an interesting investigation of the role of exact exchange to the redox potential of several ferrocene molecules. This work shows that there can be large variations in the redox potentials depending on whether pure DFT or hybrid functionals are used. Unfortunately, there are no clear rules what functional to use.

More detailed comparison of the experimental and computational data

The experimental of data redox potential for other metal than Fe is scarce. Below we have collected few computational (from the supplementary Excel file) and experimental values (from Table 1 in the main text) for comparison. The pure metal redox potentials differences when they are available. (Fe^{3+}/Fe^{2+}: 0.77 V, Cu^{2+}/Cu^{+}: 0.16 V, Ti^{4+}/Ti^{3+}: 0.0 V, Ni^{2+}/Ni^{0}: -0.23 V, Mn^{3+}/Mn^{2+}: 1.51V, Data is from Atkins, Physical Chemistry, 6 ed.)). For Cu we do not have data for metal complexes. In the case of Cu we study Cu-BIP(R1,2=H) and an average of all the Cu-BIP(R1,2=X) systems.

Metal(Ligand)	Comp/V	Exp/V	Pure metal/V
Ti(CAT)	-1.86	-1.18	0.00
Fe(CAT)	-2.05	-0.83	0.77
Difference (Fe-Ti)	+0.19	-0.35	-0.77
Mn(TEA)	-0.37	-0.24	1.51
Fe(TEA)	-0.86	-0.78	0.77
Difference (Fe-Mn)	+0.49	+0.54	0.74
Mn(EDTA)	0.39	0.84	1.51
Fe(EDTA)	0.136	0.125	0.77
Difference (Fe-Mn)	+0.25	+0.72	0.74
Ni(BIP)	2.65	1.72	
Fe(BIP)	1.12	1.08	
Difference (Fe-Ni)	+1.53	+0.64	
Cu(BIP)	-0.79		0.16
Fe(BIP)	1.12		0.77
Difference (Fe-Cu)	-1.91		-0.51
Cu(BIP-X) average	-0.64		0.16
Fe(BIP-X) average	1.22		0.77
Difference (Fe-Cu)	-1.86		-0.51

Redox potentials of different complexes

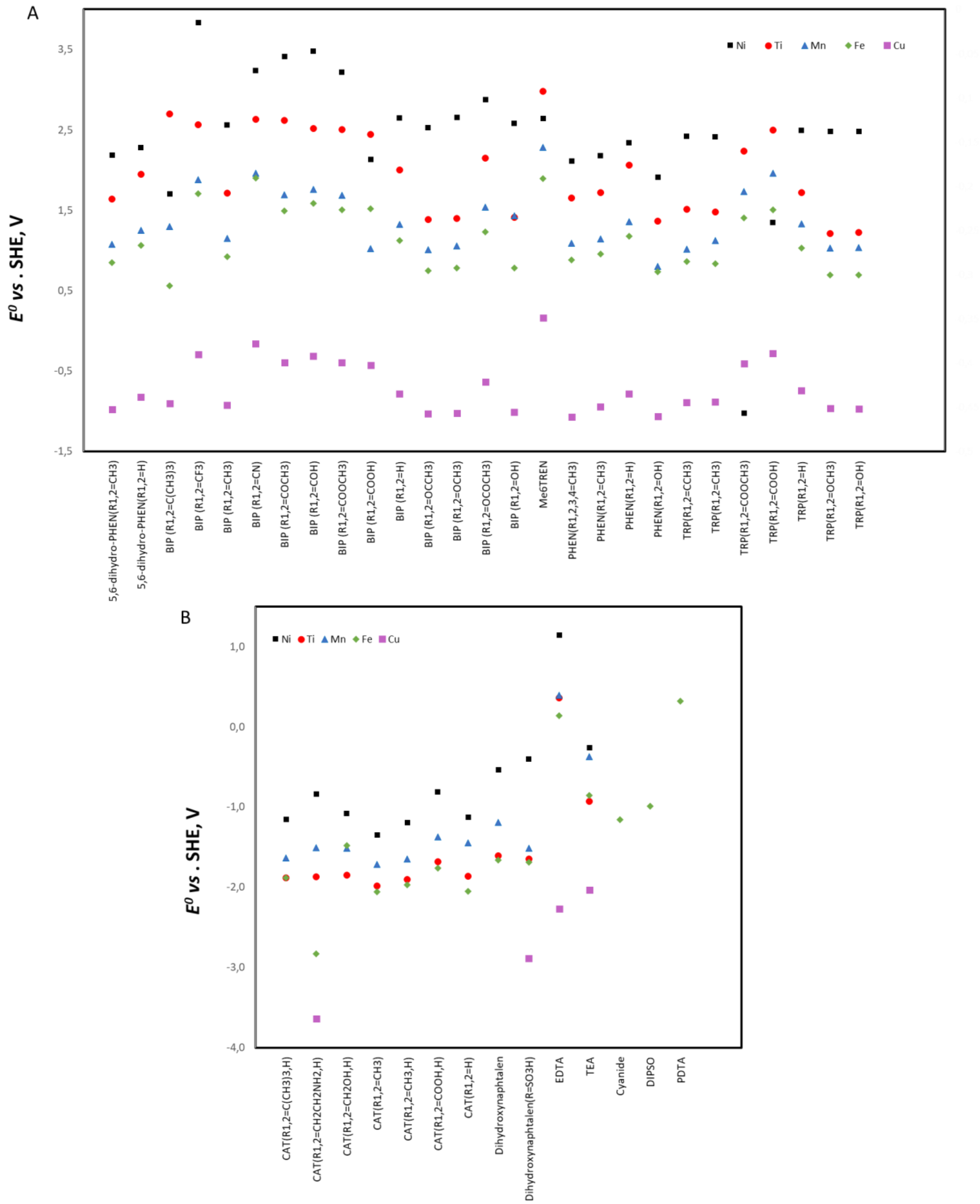

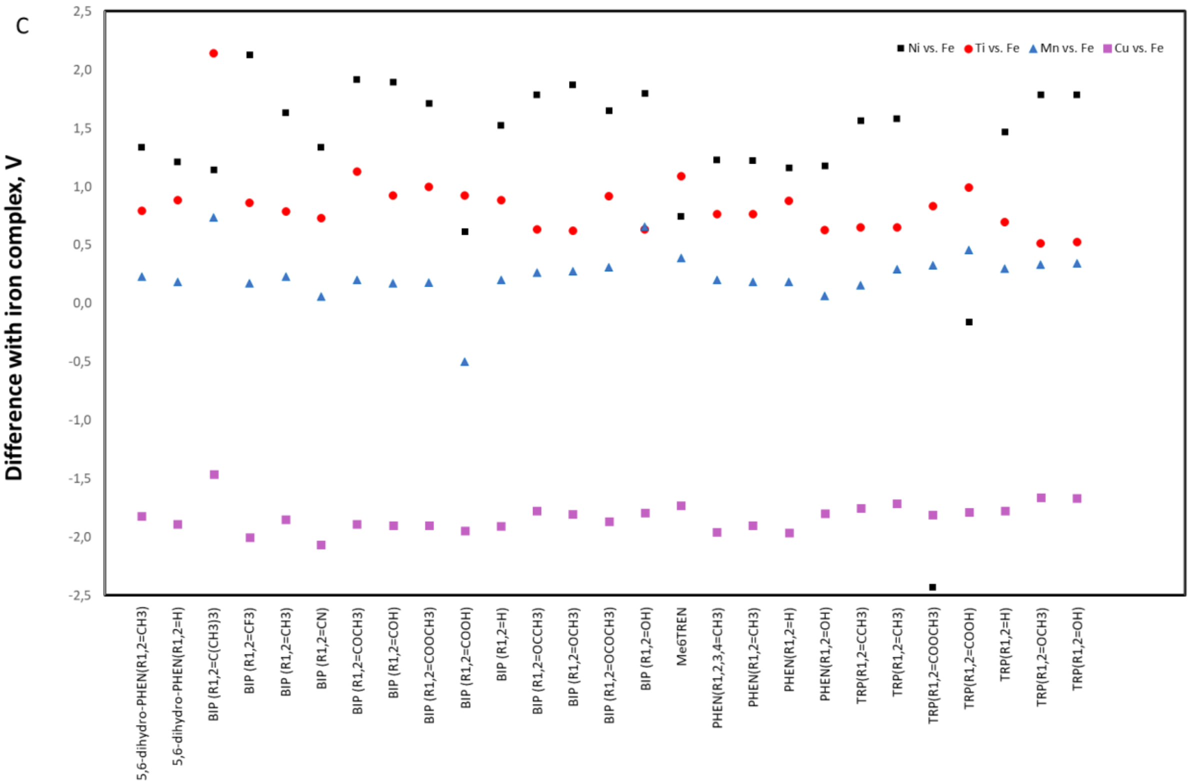
C
Difference with iron complex, V
2,5
2,0
1,5
1,0
0,5
0,0
-0,5
-1,0
-1,5
-2,0
-2,5
Ni vs. Fe
Ti vs. Fe
Mn vs. Fe
Cu vs. Fe
5,6-dihydro-PHEN(R1,2=CH3)
5,6-dihydro-PHEN(R1,2=H)
BIP (R1,2=C(CH3)3)
BIP (R1,2=CF3)
BIP (R1,2=CH3)
BIP (R1,2=CN)
BIP (R1,2=COCH3)
BIP (R1,2=COH)
BIP (R1,2=COOCH3)
BIP (R1,2=COOH)
BIP (R1,2=H)
BIP (R1,2=OCCH3)
BIP (R1,2=OCH3)
BIP (R1,2=OCOCH3)
BIP (R1,2=OH)
Me6TREN
PHEN(R1,2,3,4=CH3)
PHEN(R1,2=CH3)
PHEN(R1,2=H)
PHEN(R1,2=OH)
TRP(R1,2=CCH3)
TRP(R1,2=CH3)
TRP(R1,2=COOCH3)
TRP(R1,2=COOH)
TRP(R1,2=H)
TRP(R1,2=OCH3)
TRP(R1,2=OH)

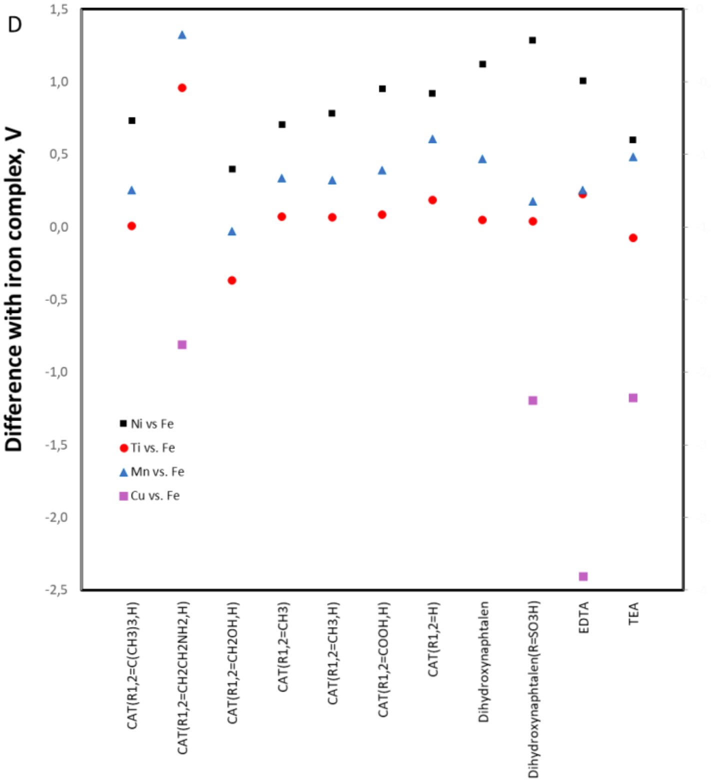
D
Difference with iron complex, V
1,5
1,0
0,5
0,0
-0,5
-1,0
-1,5
-2,0
-2,5
Ni vs Fe
Ti vs. Fe
Mn vs. Fe
Cu vs. Fe
CAT(R1,2=C(CH3)3,H)
CAT(R1,2=CH2CH2NH2,H)
CAT(R1,2=CH2OH,H)
CAT(R1,2=CH3)
CAT(R1,2=CH3,H)
CAT(R1,2=COOH,H)
CAT(R1,2=H)
Dihydroxynaphtalen
Dihydroxynaphtalen(R=SO3H)
EDTA
TEA

Figure S7. (A) The redox potential vs SHE for all complexes with N-ligands and (B) O-ligands. (C) The potential difference vs. iron complex for N-ligands and (D) O-ligands.

Solvation free energies of different complexes

The obtained theoretical solvation free energy values can be found in the supplementary excel file. Adding EWGs to the parent ligands increases, and adding the EDGs reduce the solvation free energy. The values solvation free energy of metal-BIP complexes have been given in Figure S8. Oxidized states of metal complexes including N-ligands, have higher solvation free energies than their reduced states, while the complexes with O-ligands show an opposite behavior (except complexes with central Ti metal atom). Based on the obtained results, minimum solvation is demonstrated by Cu complexes and maximum by Ti complexes. Inspection of the solvation energy results reveals the order of complex solvation energy for central metal ions to be: $\mathrm{Ti} > \mathrm{Ni} > \mathrm{Mn} \approx \mathrm{Fe} > \mathrm{Cu}$. The electrostatic attraction is the main interaction between the ions and water in the electrolyte. Therefore, the solvation of complexes with higher charge is more favorable than the complexes with lower charge in aqueous media.

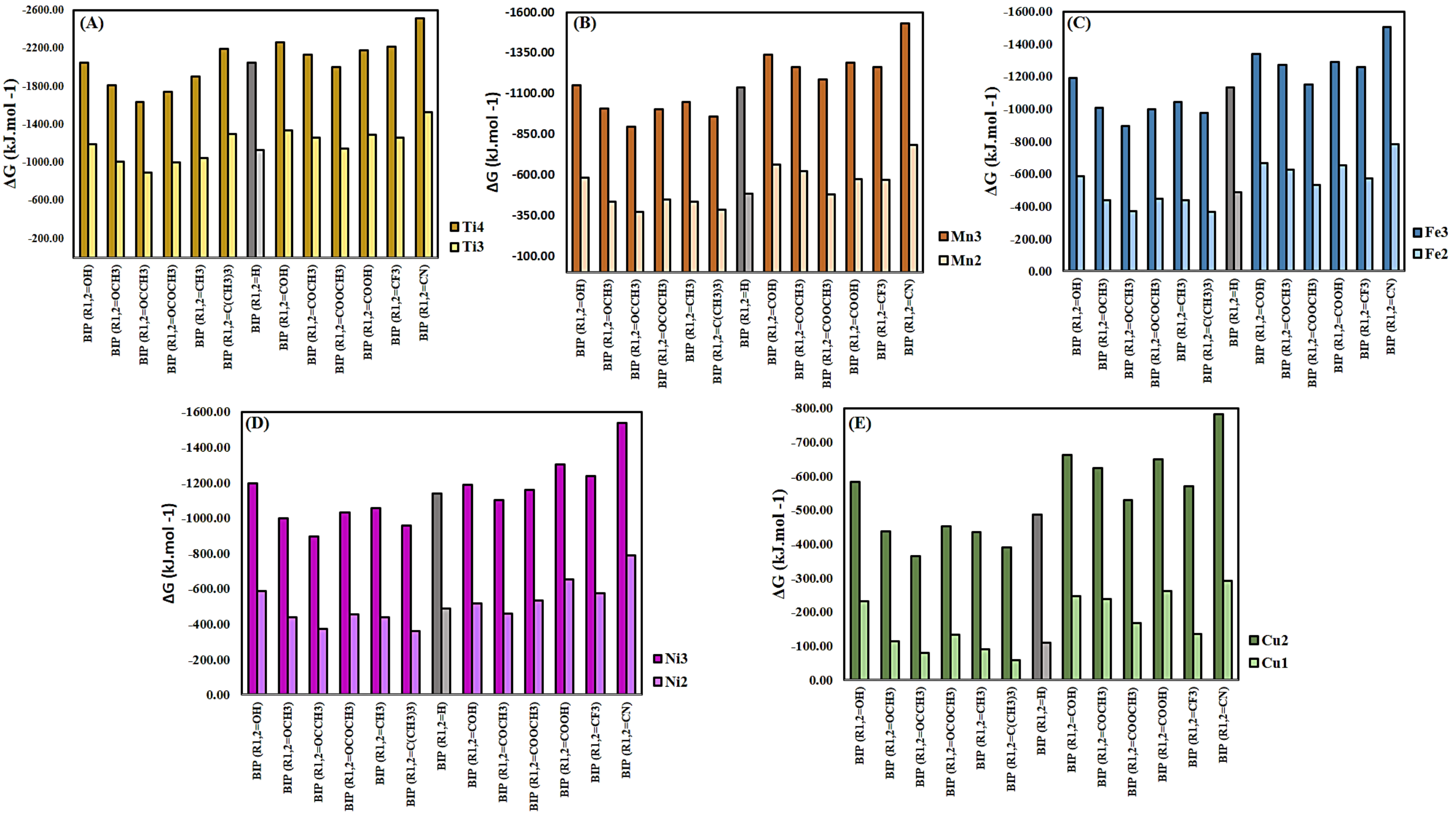

Figure S8. The solvation Gibbs energies of metal – BIP complexes family (kJ.mol^{-1}). (A) Ti, (B) Mn, (C) Fe, (D) Ni and (E) Cu.

Mapping of different complexes

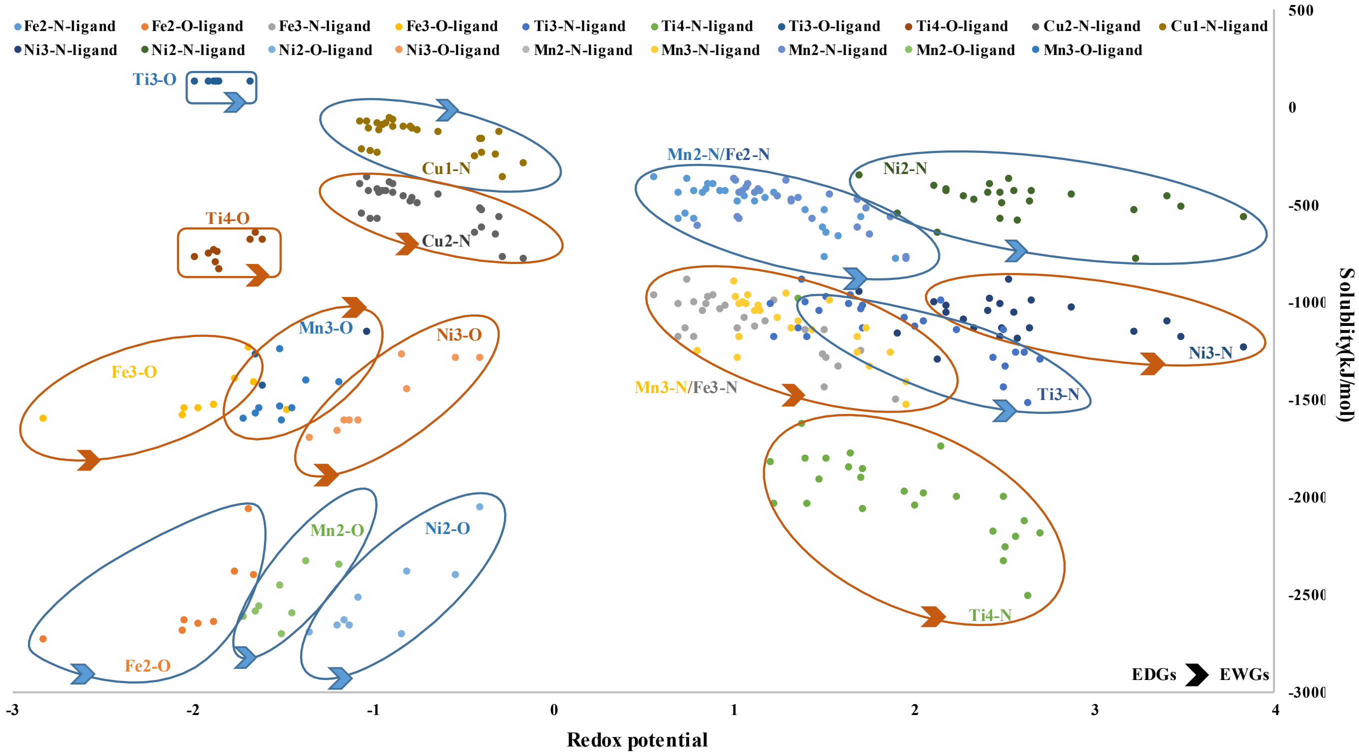

Figure S9. The redox potential vs solubility Gibbs energies of all the systems. The redox potential is between M^n and M^{n-1} so same value is used for both of them. In each cluster the arrow indicated the trend when modifying the ligand with EDG to EWG.

References

(1) Hannonen, J.; Kiesilä, A.; Mattinen, U.; Pihko, P. M.; Peljo, P. Electrochemical Characterization of Redox Activity and Stability of Various Tris(2,2‘-Bipyridine) Derived Complexes of Iron(II) in Aqueous Solutions. *Journal of Electroanalytical Chemistry* **2023**, *950*. https://doi.org/10.1016/j.jelechem.2023.117847.

(2) De La Cruz, C.; Molina, A.; Patil, N.; Ventosa, E.; Marcilla, R.; Mavrandonakis, A. New Insights into Phenazine-Based Organic Redox Flow Batteries by Using High-Throughput DFT Modelling. *Sustain Energy Fuels* **2020**, *4* (11), 5513–5521. https://doi.org/10.1039/d0se00687d.

(3) Castro, K. P.; Clikeman, T. T.; DeWeerd, N. J.; Bukovsky, E. V.; Rippy, K. C.; Kuvychko, I. V.; Hou, G.; Chen, Y.; Wang, X.; Strauss, S. H.; Boltalina, O. V. Incremental Tuning Up of Fluorous Phenazine Acceptors. *Chemistry – A European Journal* **2016**, *22* (12), 3930–3936. https://doi.org/10.1002/chem.201504122.

(4) Zhao, Y.; Truhlar, D. G. Density Functionals with Broad Applicability in Chemistry. *Acc Chem Res* **2008**, *41* (2), 157–167. https://doi.org/10.1021/ar700111a.

(5) Namazian, M.; Lin, C. Y.; Coote, M. L. Benchmark Calculations of Absolute Reduction Potential of Ferricinium/Ferrocene Couple in Nonaqueous Solutions. *J Chem Theory Comput* **2010**, *6* (9), 2721–2725. https://doi.org/10.1021/ct1003252.

(6) Grimme, S.; Antony, J.; Ehrlich, S.; Krieg, H. *J. Chem. Phys.* **2010**, *132*, 154104; DOI:10.1063/1.3382344

(7) Grimme, S.; Ehrlich, S. ; Goerigk, L. *J. Comput. Chem.* **32**, 1456 (2011); DOI:10.1002/jcc.21759

(8) Toma, M., Kuvek, T., & Vrček, V. Ionization energy and reduction potential in ferrocene derivatives: comparison of hybrid and pure DFT functionals. *The Journal of Physical Chemistry A*, **2020**, 124(39), 8029-8039.
